# Vervets revisited: A quantitative analysis of alarm call structure and context specificity

**DOI:** 10.1038/srep13220

**Published:** 2015-08-19

**Authors:** Tabitha Price, Philip Wadewitz, Dorothy Cheney, Robert Seyfarth, Kurt Hammerschmidt, Julia Fischer

**Affiliations:** 1 Cognitive Ethology Laboratory, German Primate Center, 37077 Göttingen, Germany; 2 Applied Behavioural Ecology and Ecosystem Research Unit, UNISA, South Africa; 3 Theoretical Neurophysics, MPI for Dynamics and Self-Organisation, 37077 Göttingen, Germany; 4 Department of Biology, University of Pennsylvania, Philadelphia, PA, United States; 5 Department of Psychology, University of Pennsylvania, Philadelphia, PA, United States

## Abstract

The alarm calls of vervet monkeys (*Chlorocebus pygerythrus*) constitute the classic textbook example of semantic communication in nonhuman animals, as vervet monkeys give acoustically distinct calls to different predators and these calls elicit appropriate responses in conspecifics. They also give similar sounding calls in aggressive contexts, however. Despite the central role the vervet alarm calls have played for understanding the evolution of communication, a comprehensive, quantitative analysis of the acoustic structure of these calls was lacking. We used 2-step cluster analysis to identify objective call types and discriminant function analysis to assess context specificity. Alarm calls given in response to leopards, eagles, and snakes could be well distinguished, while the inclusion of calls given in aggressive contexts yielded some overlap, specifically between female calls given to snakes, eagles and during aggression, as well as between male vervet barks (additionally recorded in South Africa) in leopard and aggressive contexts. We suggest that both cognitive appraisal of the situation and internal state contribute to the variation in call usage and structure. While the semantic properties of vervet alarm calls bear little resemblance to human words, the existing acoustic variation, possibly together with additional contextual information, allows listeners to select appropriate responses.

Language is a uniquely human trait, but a common argument is that the evolving language faculty would have been more likely to co-opt pre-existing and pre-linguistic neural and behavioural mechanisms than to evolve entirely novel language-specific cognitive modules^1^. This idea grew in prominence following the description by Struhsaker^2^ together with experiments by Seyfarth, Cheney, and Marler^3^,^4^ of the alarm calling system of the vervet monkey, *Chlorocebus pygerythrus* (previously *Cercopithecus aethiops*). These animals were reported to give acoustically distinct alarm calls to their three main predator classes. More importantly, playback experiments revealed that listeners typically selected appropriate predator avoidance behaviours even in the absence of the predator itself 3,4.

Because of their potential relevance for understanding the origins of human speech, the findings were interpreted within a linguistic framework. According to semiotic theory^5^, the relationship between a signifier and the signified can take three modes. They can be classified as indexical, when the signifier is in some way physically or causally linked to the signified, like smoke is linked to fire. In the iconic mode, the signifier bears a physical resemblance to the signified, whereas in the symbolic mode, the relationship between the signifier and the signified is arbitrary and purely conventional. Because the vervet monkey alarm calls bore no physical resemblance to the respective predator category, in the sense that they did not mimic the sounds made by the respective predators, they were deemed to be non-iconic and thus, arbitrary. As arbitrariness constitutes one of the key criteria for symbolic communication, the vervet monkey alarm calls were seen as the first example of symbolic communication in nonhuman animals. While it remained unclear whether specific alarm calls (e.g. eagle alarms) referred to a particular species of raptor, a certain escape strategy, or both^6^, it was concluded that the alarm calls of vervet monkeys designated specific external referents^4^, in a similar way as the word “table” designates a particular type of furniture. The finding that animal calls may indeed be tied to the occurrence of specific events was taken as a challenge to Darwin’s notion that animal calls merely reflect the signaller’s internal state. At the same time these findings were seen as evidence that the building blocks for semantic reference were already present in the calls of our primate ancestors.

While it was clear that listeners were able to make use of the calls to select appropriate responses, as if the calls designated specific predators, the cognitive mechanisms underlying call production remained poorly understood^6^,^7^. Macedonia and Evans^8^ pointed out that field observations and playback experiments provided only limited information about the mechanisms underlying call production. In other words, it remained unclear whether the production of alarm calls could be likened to speech production in humans, where speakers voluntarily modify the vocal output to adhere to the conventions of their respective language community, to refer to events or objects (or ideas). Irrespective of this limitation in terms of identifying the mechanisms supporting vocal production, the experiments did demonstrate that listeners responded to calls as if the calls provided information about, for example, events in the environment. Macedonia and Evans therefore coined the term “functional reference” and proposed two key criteria for classifying animal signals as functionally referential. The production criterion was that referential signals should exhibit stimulus specificity; the perception criterion was that the signal should bring about the same response as the eliciting stimuli even in the absence of supporting contextual cues^7^,^8^. The alarm, food associated, and social calls of many species of primate, other mammal, and bird have since been classified as functionally referential signals, and functional reference continues to be singled out as offering important insight into the evolution of symbolic communication in language^9^,^10^,^11^, although this view is currently debated^12^,^13^,^14^,^15^.

Because production specificity was listed as one of the two key criteria for calls that might function referentially, in the sense that they can be used by listeners to infer on-going events, one intimately related question is whether different call types are discrete or whether there are graded intermediates between call types. Barbary macaques, *Macaca sylvanus*, for instance, produce different variants of shrill barks in response to different predators, but there are no distinct boundaries between these call types^16^. Such graded variation has been found in a variety of mammal species^17^,^18^,^19^,^20^. The question of the kind of variation in acoustic structure is also of interest because it potentially provides insights into the mechanisms that underlie call production.

More than forty years have passed since Struhsaker^2^ first described the vervet alarm calls, and more than thirty years have passed since Seyfarth *et al.*’s initial playback experiments^3^. The alarm calls of vervet monkeys remain the best known and most widely cited example of semantic communication in nonhuman animals. Surprisingly, a systematic quantitative analysis of the structure of these alarm calls had never been undertaken. Previous analyses used either qualitative categorisations of alarm calls^2^,^3^ or quantitative analyses of alarm calls given only by adult females in response to snakes and eagles^21^. Yet, this analysis assessed variation in single acoustic variables, using a relatively small set of calls.

We here provide the first systematic quantitative acoustic analysis of vervet monkey calls given not only in alarm, but also aggressive contexts. We used a custom software program^22^ to extract a set of acoustic features (Table S1). We then used cluster analysis to identify objective call types among calls given in response to snakes (mostly pythons), raptors, and terrestrial predators (mostly leopards), as well as among calls given during within- and between-group aggression. From the recordings in these contexts, we used those calls that sounded similar to some of the calls given in response to predators. Specifically, we included “chutter” and “rraup”-like calls given in aggressive contexts (excluding screams), and “wrrs”, or threat grunts that also occurred in these contexts. These calls were recorded from female and male vervets from East Africa. In addition, we compared “bark”-like calls recorded from South African male vervets, which had been given in response to real and visual model leopards, and during within and between-group encounters (see also^2^). Our main interest was thus to elucidate whether “chutters”, “rraups” and “barks” given in alarm contexts would differ not only from each other but also from those given outside of alarm contexts, with the aim to achieve a better understanding of the cognitive and motivational mechanisms supporting nonhuman primate vocal behaviour. To assess how well calls could be distinguished when the context was known, we applied discriminant function analysis. Because the calls of males and females differed substantially, we ran separate analyses for the two sexes, reducing the overall variation and allowing for a clearer picture within each sex.

## Results

### Cluster analysis

**Figure 1:**
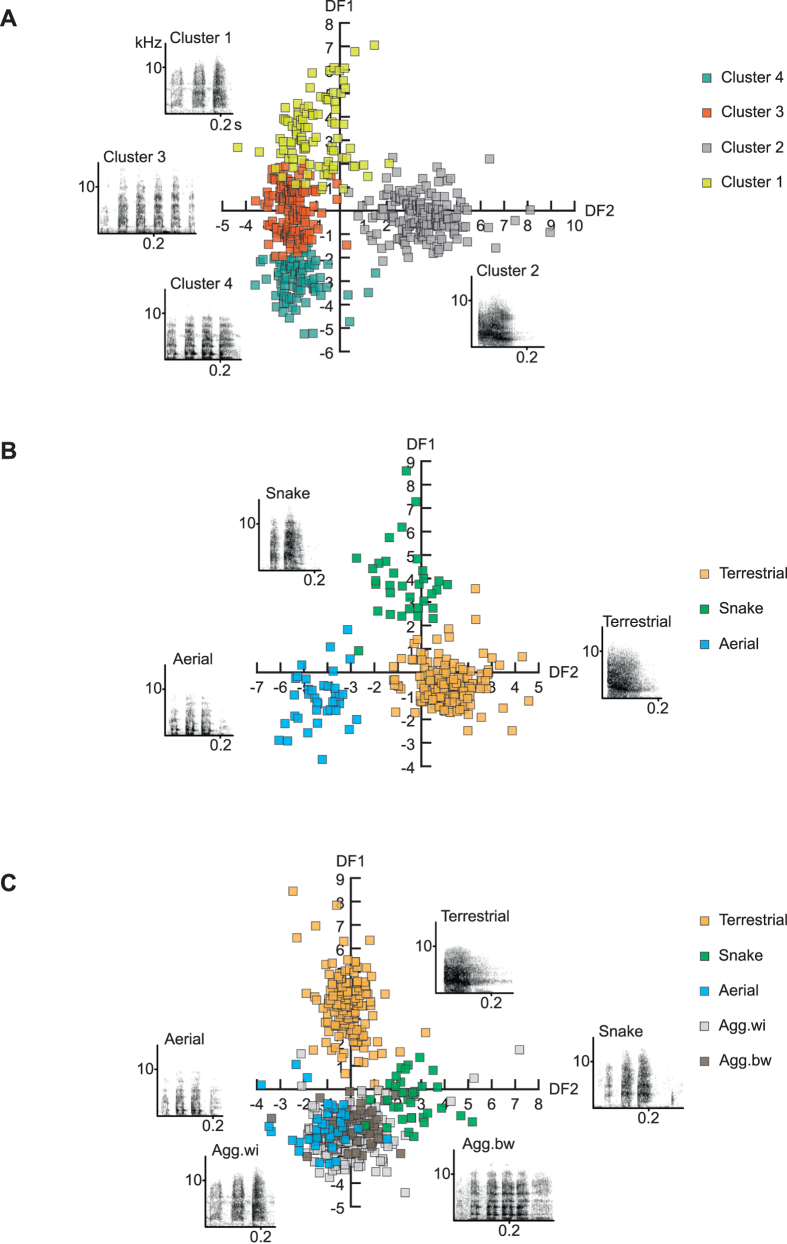
Structure and discriminability of female vervet vocalizations given in alarm and aggressive contexts. (**A**) Scatter plot of the four identified clusters based on discriminant function analyses using cluster membership as grouping variable. Spectrograms depict representative call exemplars with a small Euclidean distance to the cluster centre for each cluster. (**B**) Scatter plot of the discriminant scores with corresponding spectrograms of female alarm calls given in response to leopards, eagles, and snakes. (**C**) Scatter plot of the discriminant scores with corresponding spectrograms of female alarm calls given in response to leopards, eagles, and snakes, as well as during within- and between-group aggression. All spectrograms were made using the following settings in Avisoft: 256 FFT, frame size of 100% (Hamming window), frequency resolution 172 Hz; 50% window overlap, temporal resolution 2.9 ms. Abbreviations: kHz: Kiloherz, s: seconds, DF1: discriminant function 1, DF2: discriminant function 2.

For female East African vervet calls, the analysis identified a 4-cluster solution (Fig. 1A) with a silhouette coefficient (S_C_) = 0.5, indicating a fairly good solution. Cluster 1, 3 and 4 consisted of calls with multiple short elements, which could be distinguished based on their spectral characteristics, with cluster 1 revealing the highest frequency values, cluster 3 intermediate, and cluster 4 the lowest values. Cluster 2 consisted of calls with fewer and longer elements, and medium frequency values (see Table S2 for descriptive statistics). Calls from cluster 1 mainly occurred when the animals had spotted a snake, and during between-group aggression. Cluster 2 consisted almost exclusively of calls given during leopard encounters, while cluster 3 mainly contained calls given during between and within-group aggression. Cluster 4 encompassed calls given during between and within-group aggression, as well as in response to eagles (Table S3). The cluster solution largely matched descriptions of call types from earlier studies^2^,^3^,^4^. Cluster 1 corresponded to broadband “chutter” calls typically produced in response to snakes (usually pythons) and during intergroup encounters, cluster 2 to “chirp” calls, typically given in response to terrestrial predators (including leopards, lions, and cheetah), while cluster 4 corresponded to the low frequency “rraup” calls typically produced in response to raptors (usually martial eagles), but also during escalated between and within-group aggression. Cluster 3 fell in between cluster 1 and 4, indicating graded variation between “chutter” and “rraup” calls.

**Figure 2:**
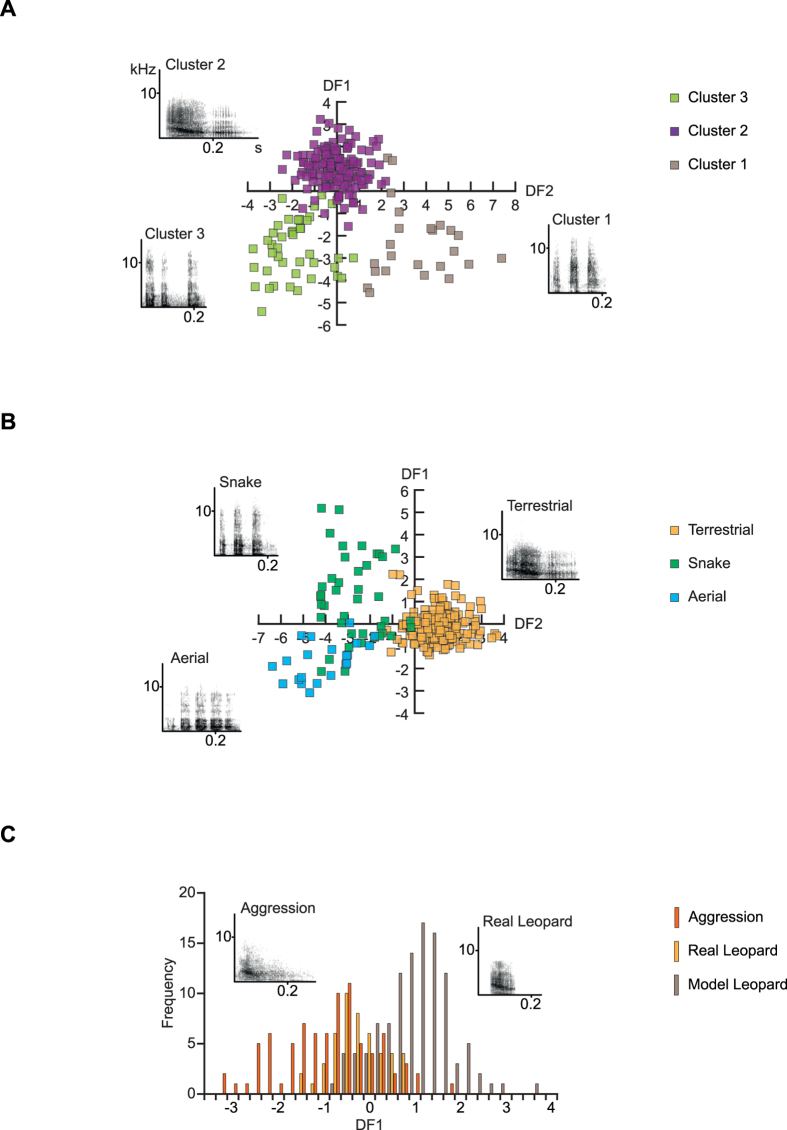
Structure and discriminability of male vervet vocalizations given in alarm and aggressive contexts. (**A**) Scatter plot of the three identified clusters based on discriminant function analyses using cluster membership as grouping variable. Spectrograms depict representative call exemplars from East African males with a small Euclidean distance to the cluster centre for each cluster. (**B**) Scatter plot of the discriminant scores with corresponding spectrograms of East African male alarm calls given in response to leopards, eagles, and snakes. (**C**) Frequency distribution of the discriminant scores with corresponding spectrograms of South African male vocalizations given in response to leopards and during within- and between-group aggression. Abbreviations as in Fig. 1.

For East African male alarm calls, the 2-step cluster procedure identified 3 clusters (Fig. 2A) with a silhouette coefficient of S_C_ = 0.5. Cluster 1 contained calls with many short elements and high frequency values, cluster 2 calls with fewer long elements and relatively high frequency values, and cluster 3 calls with a medium number of elements of medium duration, and very low frequency values (Table S4). Cluster 1 calls were mostly given in response to snakes, cluster 2 calls in response to terrestrial predators, and cluster 3 calls both in response to eagles and snakes (Table S5). Cluster 1 calls largely corresponded to earlier descriptions of male “chutters”, cluster 2 calls as “barks”, and cluster 3 as “rraup” calls^2^,^3^,^4^.

A cluster analysis of South African male vervet calls given in response to leopards, visual leopard models, and during within and between intergroup aggression revealed a 2-cluster solution as the best solution (S_C_ = 0.5). Calls in cluster 1 were characterized by higher frequency values than those in cluster 2. Overall, the clusters did not map well onto the two different contexts. Of the 101 calls assigned to cluster 1, 55 were recorded during aggressive encounters, and 46 in response to leopards. Cluster 2 calls showed a higher specificity, with 113/142 calls recorded during leopard encounters.

### Context specificity

When we considered only female calls given in the three different predator contexts, the discriminant function analysis (DFA) correctly classified 98.7% (cross-validated) of 235 calls from 24 females (Table S6). Based on the two discriminant functions, the calls could be very well distinguished (Fig. 1B). A permuted discriminant function analysis (pDFA) carried out using the same variables on a subset of the calls (N = 116 calls from 19 females) to control for individual identity revealed that this result was significantly different from chance (*P* < 0.001).

When we added calls recorded during aggressive interactions, the classification procedure (cross-validated) yielded 71.4% correct classification (Table 1). The pDFA on N = 194 calls from 25 females indicated that this was significantly different from chance (*P* < 0.001). While calls given to terrestrial predators and snakes could still be very well distinguished with an average correct classification of 94.5% and 90.9%, respectively, some misclassification occurred between the two aggression contexts and the aerial predator context. Although 81.6% of aerial predator calls were classified correctly, a number of calls given during between-group aggression were assigned to the eagle and snake contexts and some of the calls recorded during within-group aggression were assigned to the aerial predator context (Fig. 1C). Descriptive statistics of the calls given by females in the different context are shown in Table 2.

For the East African male vervet alarm calls, the DFA classified 93.2% (cross-validated) to the correct predator class (pDFA on 195 calls from 15 males: *P* < 0.002). The correct classification was high for calls given to leopards and raptors (98.3% and 85.7% respectively), while calls given to snakes were correctly classified in 75.6% of cases (Fig. 2B; Table 3). Descriptive statistics of the calls given by males in the different predator context are shown in Table 4.

Barks recorded from South African male vervet monkeys that were given in response to leopards and during intergroup aggression could be less well distinguished than calls given to different predator categories (Fig. 2C). The average correct classification of 243 calls recorded from 16 males was 74.9% (cross-validated). The classification by the pDFA on 207 calls from 16 males was only marginally better than expected by chance (*P* < 0.1). We checked whether responses to real leopards were more or less similar to calls given in the aggressive context, by comparing the distribution of discriminant scores. The inspection of the discriminant scores indicates that calls given to real leopards were more similar to calls given in aggressive context than calls given in response to model leopards (see Fig. 2C). The small sample size precluded statistical testing of this assessment.

## Discussion

East African vervet calls given in the three predator contexts were acoustically clearly discernible, confirming earlier qualitative descriptions^2^,^3^,^4^. The assessment of call specificity changed to some extent, however, when “chutter” and “rraup”-like calls from additional contexts were included in the analysis. While female calls given in response to leopards (“chirps”) were acoustically distinct from all other calls in the analysis, the inclusion of “chutter” and “rraup”-like calls from aggressive contexts yielded some graded variation between “chutters” and “rraups”, as evidenced by the continuous distribution of the calls in clusters 1, 3, and 4. Thus, clearly discernible “chutters” were given both in response to snakes and during between-group aggression^23^. Similarly, although “rraups” were given primarily in response to eagles, they also occurred during within-group aggression. Calls that fell between typical “chutters” and “rraups” occurred in both aggressive contexts. Thus, the calls of vervet monkeys given in alarm and aggressive contexts show both distinctive acoustic features (chirps) and some intergradation. In this sense, only the chirps clearly fulfil the production specificity criterion established by Macedonia and Evans^8^. Intergradation within more general call types was also found in baboons, where alarm calls grade into display and contact calls^24^, and Barbary macaque alarm calls given in different contexts^25^. Similarly, a recent study suggested that the alarm calls of Campbell’s monkeys reveal graded variation^18^, although the system was initially characterized as discrete^26^. One important caveat of this study is the possibility that we may have missed an acoustic feature that is salient to the monkeys and which would allow them to distinguish between specific variants of “chutters” and “rraups”, according to the context in which they were given. Given the ample experience with this analytical approach, specifically in combination with the results of corresponding playback experiments^22^, this does not seem to be likely, however.

What do our findings on the structure of the calls tell us about the mechanisms underlying the production of different call types? Accumulating evidence suggests that the acoustic features of calls produced by nonhuman primates is largely innate^27^ and that nonhuman primates lack direct connections from the laryngeal motor cortex to laryngeal motoneurons^28^,^29^, although individuals generally have more control over temporal aspects and the usage of calls^30^. In accordance with this, a comparison of the acoustic structure of male barks of the genus *Chlorocebus* revealed only marginal differences between calls given by two different subspecies of vervet monkeys ranging in Eastern (*C. p. pygerythrus)* and Southern Africa *(C. p. hilgerti)*, respectively, and only marginally more pronounced differences between male calls of this species and the West African congener, *C. sabaeus*^31^.

The extent to which calls reflect different cognitive, affective, and/or or motivational states of the caller remains an issue for further investigation. Clearly, the animals have rich representations of the world they live in and are able to categorize different predators, members of different social groups, and to recognize unreliable signallers^6^. Yet, their vocal repertoire is rather limited, just as in the case of other animals, such as dogs, which may understand an enormous array of different verbal commands and referents^32^, but whose vocal production is confined to a few call types such as barks and growls. Because of the contrast between the rich representation of the environment and the rather confined vocal output, one hypothesis is that different cognitive representations may elicit broadly similar affective or motivational states which in turn trigger corresponding call types. In the case of the vervet chutter, which is given in response to snakes and during between-group aggression, these internal states might reflect a degree of aversion, combined with an aggressive component^33^. Without independent evidence, the cognitive and affective/motivational/arousal components are difficult to disentangle. A study in which squirrel monkeys (*Saimiri sciureus*) could either seek or avoid electric stimulation of specific brain areas showed that these two situations predictably elicited different call types, namely shriek cackles, shrieks and alarm peeps in aversive situations, and twitters, groans, and chucks in positive contexts^34^.

Interestingly, anecdotal observations indicate that, when attacked by a stooping eagle, vervet monkeys produced “chirps” and “barks”, which would be compatible with the view that chirps and barks reflect high arousal. Yet, a number of issues remain unresolved. For instance, why do nonhuman primates give acoustically different calls in the same situation? Vervet monkeys, for example, produce at least three acoustically different calls in the context of between-group aggression. In field experiments, habituation to one call type produces habituation to another, suggesting that, by at least one measure, listeners judge the information they contain to be similar, despite their acoustic differences^35^. Similarly, captive rhesus monkeys, *Macaca mulatta*, who were tested in a habituation-recovery experiment, did not distinguish between two acoustically distinct call types that are both given to highly preferred foods^36^. Electrophysiological recordings suggested that neurons in the ventrolateral prefrontal cortex categorized the calls according to referent^37^. Another study found that the neurons were mainly sensitive to acoustic characteristics^38^, so that the question of which way the monkeys represent the calls remains an issue for further investigation.

At the ultimate level, similar motivational states may be tied to similar functions of calls. In fact, both Struhsaker^2^ and Seyfarth and colleagues^4^ identified similarities between chutters produced in alarm and contexts of within- and between-group aggression, and suggested a shared function to alert group members and solicit group defence. The loud and conspicuous bark vocalisations of adult males have been proposed as functioning to reduce the probability of a predator hunting in the vicinity again^39^, similar to the ‘perception advertisement’ hypothesis that alarm calls function to advertise predator presence^40^. Given the similarity of barks produced by males in response to leopards and during aggressive interactions, and the finding that male calling is related to rank^39^, it is possible that vervet alarm barks may also function as a sexual display advertising male fitness, akin to the “wahoo” of male baboons^41^ and the predator signals of some birds^42^,^43^,^44^. In this context, it is noteworthy that South African male vervet monkeys responded more strongly to playbacks of neighbouring male calls than to their own group males’ calls, indicating that these calls may have a function in territorial defence^31^.

Notably, males seldom called in response to snakes and raptors, possibly due to the fact that males are less vulnerable to these predators^39^. It is also interesting to note that the production of barks during within- and between-group aggression appeared to be much more common in South African than in East African males. As aggressive encounters occur frequently between vervet groups in East Africa^6^, the apparent difference in the frequency of aggression-barks suggests a population difference in call usage. Focal data would be necessary to test this assumption, and to exclude the possibility that rare incidences of call production have been missed. The visual inspection of the distribution of discriminant function scores (Fig. 2C) further indicates that the intergradation of calls given in the two contexts is not due to the inclusion of calls given in response to leopard models. In fact, the calls given in response to the models appeared to be more distinct from calls given during aggression that calls given in response to real leopards. The intergradation is therefore not simply an artefact of the recording conditions.

Earlier studies described animal vocalisations as falling along a continuum; from calls primarily reflecting the signaller’s motivational state to calls reliably elicited by an external stimulus and therefore providing information in addition to the signaller’s motivational state^8^. From the listener’s perspective, the source of acoustic variation is of only secondary importance. Vocal cues may be associated with the caller’s affective state, identity, behaviour, and/or an external stimulus, and the cognitive mechanisms underlying responses are likely no different when responding to these different cues, or indeed to other sources of information. Thus, affect-based calls may also meet the requirements of so-called functionally referential signals, if the recipient is able to interpret these signals to make inferences about external events^7^,^45^. Viewed in this way, the dichotomy between affect-based and referential calls disappears; call production may or may not be related to the caller’s internal state, and any similarity with true referential communication is restricted to how calls are perceived^46^. Accordingly, the identification of “functionally referential” signals does not offer substantial insights into the evolution of semantic reference in human speech production^12^,^46^.

Characterizing a system as graded does not entail that listeners are unable to retrieve information about the context that elicited the calling. As mentioned above, Barbary macaques are able to categorise graded acoustic variation into different meaningful categories^16^. Thus, the identification of graded overlap between calls does not necessarily imply that listeners are unable to identify different categories. Indeed, members of a wide variety of taxa, including birds and frogs, show categorical perception of certain call types^47^,^48^,^49^. In addition to acoustic variation, contextual cues can be used to clarify what a call stands for. For instance, it may be that in the absence of any other cues indicating a fight between two group members, a vervet will tend to judge a “rraup” call as an eagle alarm. Indeed, in Seyfarth *et al.*’s original playback experiments^4^, subjects consistently looked toward the source of the call – as if searching for additional contextual information – in addition to showing predator-specific responses. Playback experiments that systematically vary contextual and acoustic information will be needed to examine this conjecture. Notably, when West African green monkeys were primed with either the presence of a snake or a leopard, their initial responses to ambiguous alarm chirps did not differ substantially, while their long-term responses did^50^. Similarly, putty nosed monkeys (*Cercopithecus nictitans*) used contextual information to disambiguate calls that revealed graded variation^51^. The integration of information retrieved from ambiguous signal and context may, in fact, present a more complex cognitive challenge to receivers compared to the interpretation of highly context-specific calls alone^12^. To determine the cognitive load associated with the interpretation of signals, information about true context specificity is needed. Strictly speaking, for this, all calls from all contexts need to be sampled, while comparisons of calls given in a limited number of contexts likely overestimate context specificity^52^.

Future studies should consider how animals make judgments and decisions under uncertainty when call structure is ambiguous^53^, and how they integrate additional information such as caller identity and contextual information to select appropriate responses. In conclusion, while the field of nonhuman primate semantics has revealed fundamental differences in the mechanisms underlying call production in nonhuman primates and humans, greater commonalities can be found in the field of primate pragmatics^54^. More broadly, we believe it is now time to move beyond the metaphor of functionally referential alarm calls as precursors to symbolic communication^13^, and focus instead on the selective pressures shaping primate vocalizations over evolutionary time.

## Methods

### Data collection

Vocalisations of East African vervets were recorded in analogue form from several free-ranging habituated groups within Amboseli National Park in Kenya, by T. Struhsaker (June 1963-May 1964) and subsequently by R. M. Seyfarth and D. L. Cheney (1977–1988). These calls were digitised post-recording at 44.1 and 22.05 kHz and 16 bit sampling depth. For a more detailed description of study subjects, study sites and recording equipment see^2^,^4^. South African vervet vocalisations were recorded by T. Price (Jan-June 2012), using a Marantz PMD661 solid-state recorder (44.1 kHz sampling rate; 16-bit sampling depth) connected to a Sennheiser ME66/K6 directional microphone from four free-ranging habituated groups within the Loskop Dam Nature Reserve in South Africa. Spontaneous vocalisations were recorded using focal individual and/or ad libitum sampling techniques during all contributing studies, and R. Seyfarth, D. Cheney and T. Price supplemented recordings elicited by snakes and leopards with vocalisations given in response to visual snake and leopard models. Recordings of vervet vocalizations were in accordance with the approved guidelines by the Office of the President of the Republic of Kenya and the Mpumalanga Parks Board of South Africa.

### Call selection

To assess the acoustic structure of East African vervet alarm calls and the degree to which they are predator-specific, all vocalisations that were recorded in the presence of a raptor, leopard or snake were selected. Calls given by immature and unidentified individuals were discarded to avoid age-related effects and to control for individual differences in analyses. Spectrograms of vocalisations were inspected visually using Avisoft-SASLab Pro (R. Specht Berlin, Germany, version 5.1.20); calls were selected for analysis when they were undisturbed by other sounds and possessed a high signal-to-noise ratio. To assess the degree to which vervet alarm calls are context-specific, we next incorporated calls produced outside of the predator context into our analyses. For females we included calls given during within- and between-group aggression recorded in East Africa that resembled calls given in alarm contexts (“chutter” and “rraup”-like calls). Vervet monkeys also produce screams, threat-grunts and so-called “wrrr” calls in these contexts; these were not considered^35^. Whilst East African adult male vervets produce barks in contexts of aggression as well as in response to terrestrial and aerial predators^2^,^6^, there were insufficient recordings of these calls from this population to allow for a structural comparison between contexts. Instead, we included a separate analysis of male vervet calls produced in contexts of aggression (within- and between-group chases, physical attacks and between-group confrontations) and in response to leopards and leopard models in South Africa. The South African leopard alarms were recorded from 10 individuals with the calls of each individual produced within a different calling event. Three of these calling events were recorded in response to real leopards (once the leopard was seen by researchers and twice it was identified from hearing its growls nearby in the undergrowth without researchers being able to see it); the seven other calling events occurred in response to model presentation. In some contexts individuals produced very long calling bouts consisting of hundreds of calls; to get a more balanced sample for spectral analysis of East and South African recordings, we selected a maximum of 16 calls from any one calling bout. The final data set comprised a total of 504 calls from 37 females, 237 calls from 15 E. African and 243 calls from 16 S. African males.

### Acoustic analysis

The majority of vervet vocalizations given in alarm and aggressive contexts are comprised of multiple elements, so we measured temporal features on the compound call, including overall call duration, number of elements, and inter-call interval. Spectral features were determined from the single elements (see Table S1 for details). Firstly, we used Avisoft -SASLab Pro to filter recordings (high-band filter set at 0.1 kHz) to remove background noise below the lowest frequency of calls. The start and end points of all call elements were labelled and these measures were exported for the calculation of temporal measures. We then determined the “main element”, i.e. the element with the highest amplitude, and the remaining elements. All elements were padded with silent margins and extracted for spectral analysis. No calls contained energy above 11 kHz, so we reduced the sampling frequency of all calls to 22.05 kHz in order to standardise the sampling frequency of calls within each data set and to optimise the frequency resolution.

Call elements were transformed in their frequency-time domain with Avisoft using a fast Fourier transform (FFT) size of 1024 points with Hamming window and 93.75% overlap. This resulted in bandwidths of 28 Hz and a time step of 2.9 ms. The resulting frequency-time spectra were analysed with LMA (Lautmusteranalyse) v. 2012_9, a custom software sound analysis tool developed by K. Hammerschmidt. LMA allows for the extraction of a wide range of robust acoustic variables^22^. Because many of the calls in the data set lack harmonic structure, we refrained from determining the fundamental frequency, and chose instead variables that describe the energy distribution throughout the call. Avisoft was used to extract element duration from the wav file, and to calculate Wiener entropy. For the main element of a given call, we determined a suite of acoustic variables. In addition, we determined the mean of the mean peak frequency, the mean second quartile of the distribution of frequency amplitudes, and the mean element duration for all of the elements in the call, so that each call could be described in terms of the temporal characteristics, the spectral characteristics of the main element, and the average characteristics of all elements (see SI).

### Statistical analysis

To identify the structure of the calls without prior knowledge about the context, we used 2-step cluster analyses on 10 selected acoustic variables (see Table S1 for the rationale of parameter selection). We used the Schwarz’s Bayes’ Information Criterion (BIC), log-likelihood as distance measure, and a maximum of 15 potential clusters to identify the most appropriate cluster solution. To determine whether calls given in different contexts are distinguishable we ran discriminant function analyses. We entered all independents together, and calculated the average classification using the jack-knifed (i.e., leave one out) procedure. The cluster analyses and conventional DFA analyses were done using IBM SPSS 21. Because DFA classification is sensitive to unbalanced samples, we additionally ran nested permuted discriminant function analyses^55^ on a subset of the data using the same variables, which allowed us to control for caller identity. In adherence to the requirements of nested pDFAs, calls from a single individual were selected only from one level of the test factor, and only if three or more calls were available from within this single level. For the pDFA, we used a function written by Roger Mundry in R^56^ on z-transformed variables. The function is based on the function lda of the R package MASS^57^.

## Additional Information

**How to cite this article**: Price, T. *et al.* Vervets revisited: A quantitative analysis of alarm call structure and context specificity. *Sci. Rep.* **5**, 13220; doi: 10.1038/srep13220 (2015).

## Supplementary Material

Supplementary Information

## Figures and Tables

**Table 1:** Classification Results of the discriminant function analysis for female calls recorded in alarm and aggression contexts.

	Context	Predicted Group Membership					Total
		**Agg. between**	**Agg. within**	**Aerial**	**Snake**	**Terrestrial**	
Count	Agg. between	119	49	37	10	1	216
	Agg. within	14	25	11	3	0	53
	Eagle	1	6	31	0	0	38
	Snake	0	3	0	30	0	33
	Terrestrial	0	0	1	8	155	164
%	Agg. between	55.1	22.7	17.1	4.6	0.5	100.0
	Agg. within	26.4	47.2	20.8	5.7	0.0	100.0
	Eagle	2.6	15.8	81.6	0.0	0.0	100.0
	Snake	0.0	9.1	0.0	90.9	0.0	100.0
	Terrestrial	0.0	0.0	0.6	4.9	94.5	100.0

**Table 2:** Descriptive statistics for female calls given in alarm and aggressive contexts (N = 504 calls).

	Context									
	Aggression between		Aggression within		Aerial		Snake		Terrestrial	
	Mean	SEM	Mean	SEM	Mean	SEM	Mean	SEM	Mean	SEM
Number of elements	4.3	0.1	3.4	0.2	2.7	0.1	4.1	0.3	1.4	0.1
Element duration [ms]	38.9	0.7	39.1	1.0	43.9	2.8	38.3	1.3	107.7	2.3
Wiener Entropy	0.68	0.01	0.66	0.01	0.53	0.02	0.72	0.01	0.62	0.01
Peak frequency [Hz]	1715	90	1456	140	986	54	2840	115	2374	31
First quartile [Hz]	1707	48	1559	77	990	47	2634	71	2224	25
Second quartile [Hz]	3402	92	2871	124	1743	100	3867	103	2981	37
PF jump [Hz]	2106	150	1617	174	686	104	1588	215	1085	64
Frequency range [Hz]	7048	171	6126	268	3793	310	7162	198	4092	80
FP1 mean [Hz]	1306	57	1084	101	933	53	1640	157	2207	34
FP1A mean [rel. Amp.]	509	13	527	26	659	38	450	29	1233	29

**Table 3:** Classification results of the discriminant function analysis for male alarm calls.

	Context	Predicted Group Membership			Total
		Aerial	Snake	Terrestrial	
Count	Eagle	18	3	0	21
	Snake	7	31	3	41
	Terrestrial	0	3	172	175
%	Eagle	85.7	14.3	0	100.0
	Snake	17.1	75.6	7.3	100.0
	Terrestrial	0	1.7	98.3	100.0

**Table 4:** Descriptive statistics for male calls given in alarm contexts (N = 237 calls).

	Context					
	Aerial		Snake		Terrestrial	
	Mean	SEM	Mean	SEM	Mean	SEM
Number of elements	4.2	0.4	5.1	0.6	3.1	0.2
Element duration	76.5	4.6	61.1	4.6	112.0	2.0
Wiener Entropy	0.37	0.02	0.55	0.01	0.60	0.01
Peak frequency [Hz]	1090	88	1639	118	1904	22
First quartile [Hz]	916	60	1435	82	1753	18
Second quartile [Hz]	1311	54	2282	116	2393	24
PF jump [Hz]	543	94	1503	169	654	35
Frequency range [Hz]	1832	99	4069	264	3794	47
FP1 mean [Hz]	1048	98	1248	92	1776	27
FP1A mean [rel. Amp.]	1278	55	784	79	819	14
