# Supplementary material for: Vervets revisited: A quantitative analysis of alarm call structure and context specificity
Source: Sci Rep. 2015 Aug 19;5:13220. doi: 10.1038/srep13220 (PMC4541072; doi:10.1038/srep13220)
Supplement: Supplementary Information [file srep13220-s1.pdf]

Supplementary Information

Vervets revisited: A quantitative analysis of alarm call structure and context specificity

Tabitha Price[1,2], Philip Wadewitz[1,3], Dorothy Cheney[4], Robert Seyfarth[5], Kurt Hammerschmidt[1] and Julia Fischer[1,*]

Selection of Acoustic variables

Because a number of the acoustic variables were highly correlating, we first used stepwise discriminant function analysis to reduce the set of variables needed to differentiate between contexts. The selection criterion for an acoustic parameter to be entered was F = 3.84 and F = 2.71 to be removed from the analysis. For females, this yielded a total of 8 variables; for males, an additional 3 variables were needed. As the number of variables may strongly influence the outcome of cluster analyses, and we wanted to keep the analyses of male and female calls as similar as possible, we settled on a final set of 10 variables for both sexes (Tab. S1). Nevertheless, we conducted all analyses for males and females separately.

Table S1. Description of Acoustic Variables that were initially calculated and indication of variables selected based on a step-wise discriminant function analysis. Variables shown in bold are used in the cluster and discriminant function analysis.

Acoustic Variable	Description	Extracted for females	Extracted for males
Temporal call measures			
Call duration (ms)	Duration of call		
**Number of elements**	Number of call elements within a call	X	X
Inter-element interval (ms)	Mean inter-element interval within a call		
Element measures			
**Element duration (ms)**	Duration of call element	X	X
**Peak frequency (Hz)**	Mean peak frequency	X	X
**PF jump (Hz)**	Maximum difference between successive peak frequencies		X
PF linear trend (-1 to 1)	Factor of linear trend of peak frequency throughout the call		
PF deviation (Hz)	Mean deviation between peak frequency and linear trend		
**First quartile (Hz)**	Mean first quartile of amplitude in spectrum	X	X
**Second quartile (Hz)**	Mean second quartile of amplitude in spectrum		X
**FP1 mean (Hz)**	Mean frequency of the first global frequency peak	X	X
**FP1A mean (rel. amplitude)**	Mean amplitude of the first global frequency peak	X	X
Dominant frequency (Hz)	Mean dominant frequency		X
**Frequency range (Hz)**	Mean frequency range	X	X
**Wiener Entropy**	Mean value of noise within call. 0=pure tone, 1= random noise	X	X
Average element measures			
Mean second quartile (Hz)	see above – across all elements in call		
Mean element duration (ms)	see above – across all elements in call		
Mean peak frequency (Hz)	see above – across all elements in call		

Table S2. Descriptive statistics (mean and SEM) for the four clusters identified for female calls.

	Cluster Number							
	1		2		3		4	
Acoustic Variable	Mean	SEM	Mean	SEM	Mean	SEM	Mean	SEM
Number of elements	4.13	0.19	1.43	0.06	4.07	0.17	3.47	0.17
Element duration	38.4	1.1	108.5	2.3	38.5	0.7	42.8	1.5
Wiener Entropy	0.78	0.01	0.61	0.01	0.69	0.01	0.51	0.01
Peak frequency	3137	149	2367	31	1298	40	953	37
First quartile	2601	65	2220	25	1574	24	1009	24
Second quartile	4712	89	2964	36	3166	50	1704	40
PF jump	3926	231	1045	60	1228	89	721	55
Frequency range	8883	145	4051	78	7075	116	3407	103
FP1 mean	2045	105	2193	33	1065	39	853	39
FP1A mean	380	15	1248	28	516	12	672	22

Table S3. Distribution of female calls recorded in the different contexts across the four clusters identified by the 2-step cluster analysis.

		Cluster Number				Total
		1 „chutter“	2 „chirp“	3 „rraup“	4 „chutter-rraup“	
context	Agg. between	62	0	105	49	216
	Agg. within	6	0	32	15	53
	Eagle	0	0	6	32	38
	Snake	24	1	8	0	33
	Terrestrial	3	160	1	0	164
Total		95	161	152	96	504

Table S4. Descriptive statistics (mean and SEM) for the three clusters identified for male calls.

	Cluster Number					
	1		2		3	
Acoustic Variable	Mean	SEM	Mean	SEM	Mean	SEM
Number of elements	6.21	0.79	3.05	0.17	4.17	0.42
Element duration	46.4	4.8	112.2	2.1	81.3	3.6
Wiener Entropy	0.62	0.02	0.60	0.00	0.43	0.01
Peak frequency	1943	168	1909	20	1195	69
First quartile	1772	104	1753	18	1015	43
Second quartile	2825	121	2394	23	1493	47
PF jump	1924	174	633	33	787	132
Frequency range	5205	276	3799	44	2255	114
FP1 mean	1293	143	1784	26	1140	74
FP1A mean	435	29	817	14	1240	55

Table S5. Distribution of male calls recorded in the different contexts across the three clusters identified by the 2-step cluster analysis.

		Cluster Number			Total
		1 „chutter“	2 „bark“	3 „rraup“	
	Eagle	0	0	21	21
Subcontext	Snake	20	3	18	41
	Terrestrial	4	168	3	175
Total		24	171	42	237

Table S6. Classification Results of the discriminant function analysis for female alarm calls.

	Context	Predicted Group Membership			Total
		Eagle	Snake	Terrestrial	
	Eagle	37	1	0	38
Count	Snake	1	32	0	33
	Terrestrial	0	1	163	164
	Eagle	97.4	2.6	.0	100.0
%	Snake	3.0	97.0	.0	100.0
	Terrestrial	.0	.6	99.4	100.0
